# MosaiCatcher v2: a single-cell structural variations detection and analysis reference framework based on Strand-seq

**DOI:** 10.1093/bioinformatics/btad633

**Published:** 2023-10-18

**Authors:** Thomas Weber, Marco Raffaele Cosenza, Jan Korbel

**Affiliations:** European Molecular Biology Laboratory, Genome Biology Unit, Heidelberg, Germany; European Molecular Biology Laboratory, Genome Biology Unit, Heidelberg, Germany; European Molecular Biology Laboratory, Genome Biology Unit, Heidelberg, Germany; Bridging Research Division on Mechanisms of Genomic Variation and Data Science, German Cancer Research Center (DKFZ), Heidelberg, Germany

## Abstract

**Summary:**

Single-cell DNA template strand sequencing (Strand-seq) allows a range of various genomic analysis including chromosome length haplotype phasing and structural variation (SV) calling in individual cells. Here, we present MosaiCatcher v2, a standardized workflow and reference framework for single-cell SV detection using Strand-seq. This framework introduces a range of functionalities, including: an automated upstream Quality Control (QC) and assembly sub-workflow that relies on multiple genome assemblies and incorporates a multistep normalization module, integration of the single-cell nucleosome occupancy and genetic variation analysis SV functional characterization and of the ArbiGent SV genotyping modules, platform portability, as well as a user-friendly and shareable web report. These new features of MosaiCatcher v2 enable reproducible computational processing of Strand-seq data, which are increasingly used in human genetics and single-cell genomics, toward production environments. MosaiCatcher v2 is compatible with both container and conda environments, ensuring reproducibility and robustness and positioning the framework as a cornerstone in computational processing of Strand-seq data.

**Availability and implementation:**

MosaiCatcher v2 is a standardized workflow, implemented using the Snakemake workflow management system. The pipeline is available on GitHub: https://github.com/friendsofstrandseq/mosaicatcher-pipeline/ and on the snakemake-workflow-catalog: https://snakemake.github.io/snakemake-workflow-catalog/?usage=friendsofstrandseq/mosaicatcher-pipeline. Strand-seq example input data used in the publication can be found in the Data availability statement. Additionally, a lightweight dataset for test purposes can be found on the GitHub repository.

## 1 Introduction

Strand-seq is an amplification-free single-cell short-read sequencing technique that generates strand-specific libraries by targeting and sequencing specifically template strand during DNA replication (Falconer *et al.* 2012). Strand-seq methodology enables diverse applications not readily accessible to other technologies: characterization of a wide variety of SV classes in single cells including balanced inversions as well as complex genomic variation such as chromothripsis events (Sanders *et al.* 2016, Porubsky *et al.* 2022), locating sister chromatid exchange events (Claussin *et al.* 2017), single cell multi-omic analyses linking genome and molecular phenotype in the same cell (Jeong *et al.* 2023), generation of long-range phase information to assist genome assembly (Ebert *et al.* 2021, Porubsky *et al.* 2021, 2022, Jarvis *et al.* 2022), contiguity validation of long-range assemblies (Nurk *et al.* 2022), and characterization of somatic SV events at the single-cell level, and thus in subclonal cell populations, to foster studies in cancer evolution (Sanders *et al.* 2020, Jeong *et al.* 2023).

Regarding this latter application, a computational framework, named MosaiCatcher was developed alongside the wet lab methodology in order to process Strand-seq data, based on a tri-channel processing implementation including three layers of information: depth of coverage, strand directionality, and haplotype phase (Sanders *et al.* 2020).

Compared to SV calling using bulk-tissue whole-genome sequencing (WGS), MosaiCatcher offers a unique lens to view SVs at the single-cell individual level. While the SV breakpoint calling resolution is ∼100 kb due to read binning when analyzing SVs (Sanders *et al.* 2020), Strand-seq unveils heterogeneities often masked in bulk tissue samples and enables the detection of haplotype-phased SV. Understanding these methodological aspects, especially when contrasting bulk-tissue WGS with single-cell Strand-seq, is pivotal to comprehend for an accurate analysis of the data.

Here, we present MosaiCatcher v2, addressing both prior technical limitations and providing a range of new functionalities including: an upstream QC and assembly sub-pipeline that can rely on the most recent human genome assemblies (hg19, hg38, T2T-CHM13), as well as on mouse genome assembly (mm10) and a new multistep normalization module. Additionally, MosaiCatcher v2 incorporates a structural variation (SV) functional analysis module, which uses nucleosome occupancy data measured directly from Strand-seq libraries (single-cell nucleosome occupancy and genetic variation analysis, scNOVA) as well as a SV genotyper (ArbiGent). This updated version also offers increased stability through data-conditional dependent execution, and a sharable user-friendly HTML web report including all the relevant outputs, statistics, and plots computed during the workflow execution. MosaiCatcher v2 is a standardized, stable, and portable workflow designed to serve as a core pipeline for researchers working with Strand-seq data. Our primary goal here is to streamline and enhance the efficiency of processing Strand-seq data for the scientific community. By incorporating the latest advancements, MosaiCatcher v2 not only offers an easy-to-use pipeline but also ensures compatibility with high-scale production environments, thus enabling researchers to tackle large-scale studies and accommodate the growing volume of Strand-seq data using a comprehensive, integrated system.

## 2 Features

**Figure 1.**
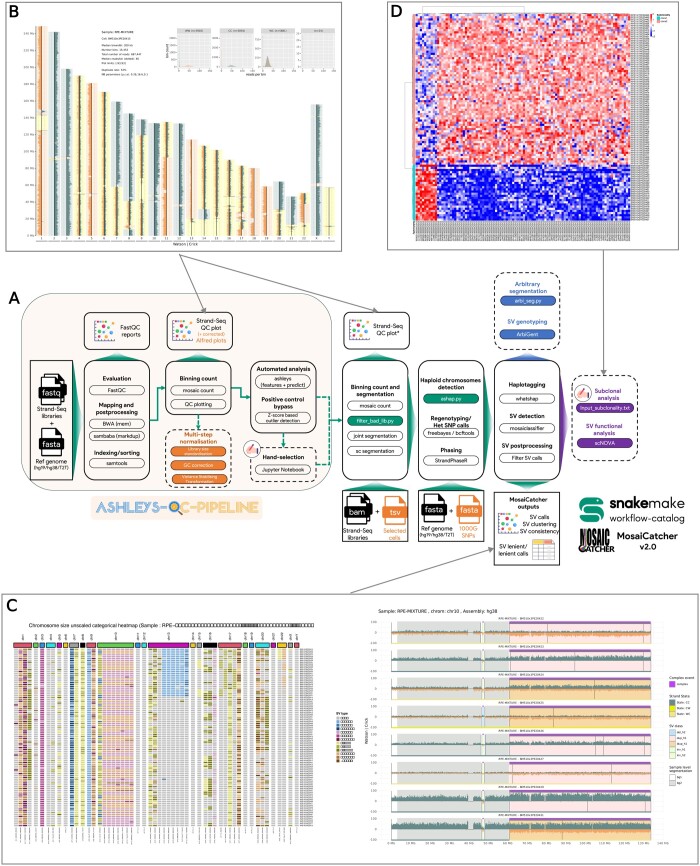
MosaiCatcher v2 schematic representation and visualizations examples. (**A**) MosaiCatcher v2 pipeline schematic representation: On the left part in dimmed orange is represented ashleys-qc-pipeline, a switchable preprocessing optional module that allows to perform standard steps of mapping, sorting, and indexing FASTQ libraries, producing quality control plots and reports as well as identifying high-quality libraries. On the right uncolored part, the MosaiCatcher core part of the pipeline is still usable as a standalone by providing Stand-Seq aligned BAM files. Green boxes correspond to data-conditional dependent execution steps (Snakemake checkpoints) that allow more flexibility and reduce issues when executing the workflow. Orange box corresponds to the multi-step normalization module. Blue box corresponds to ArbiGent mode of execution that allows SV genotyping from arbitrary segmentation. Violet box corresponds to scNOVA SV function analysis mode of execution. Dashed boxes correspond to optional modules. *: Strand-seq QC plot is only produced here if ashleys-qc pipeline is not enabled (**B**) quality control Strand-seq karyotype visualization based on read counting according to a defined window (here 200 kb). A karyotype plot is available for each single-cell sequenced. Additional statistics are also presented in the upper part of the figure. These plots allow the users to perform QC to assess sequencing integrity. (**C**) SV call clustering heatmap and chromosome-wise visualizations. The clustering heatmap on the left generates a global SV distribution representation of the sample. Each SV call is represented as a column and each library as a row. Top row annotation corresponds to chromosomes and individual heatmap cell color corresponds to a specific type of haplotype-phased SV defined in the legend. The chromosome-wise representation on the right side of the subfigure summarizes, for a given chromosome (here chr10), the different layers of information computed during the pipeline: binned read counts, joint and cell segmentation, and phased SV calls. (**D**) Differential nucleosome occupancy heatmap representation: this heatmap computed with the scNOVA downstream module exemplifies gene-body-based differential nucleosome occupancy profiles (Jeong *et al.* 2023).

MosaiCatcher v2, as its prior release, relies on Snakemake (Mölder *et al.* 2021), a workflow management system widely adopted in the bioinformatics community. Snakemake allows portability and scalability to almost all existing cluster and cloud platforms based on conda environments and containers. With the aim of standardizing Strand-seq data analysis and promoting the dissemination of this technology, MosaiCatcher v2 (schematically represented in Fig. 1) we have improved stability and integrated new functionalities (listed below and detailed in the Supplementary Material):

Strand-seq end-to-end processing and analysis: the previous version of MosaiCatcher relied on cumbersome manual preprocessing and filtering of high-quality libraries. To address this issue, the ashleys-qc (Gros *et al.* 2021) machine-learning-based tool was developed to automatically select high-quality libraries. In this new MosaiCatcher version, ashleys-qc is seamlessly integrated, enabling end-to-end computational processing from the raw sequencing FASTQ data to the downstream analysis including single-cell SV calling and visualization analysis, thus promoting reproducibility and reducing bias in the analysis. This is achieved using data-dependent conditional execution steps (see below) that lead to the selection and the processing of high-quality labeled libraries through ashleys-qc. However, if needed, manual filtering by a domain-expert is still possible through a switchable option that gives to the user the possibility to identify and flag the high-quality libraries through a jupyter notebook. The ashleys-qc-pipeline was cleaned and formatted to be referenced as a standardized usage pipeline on the snakemake-workflow-catalog and usable as a module, loadable in MosaiCatcher v2.

**Data-dependent conditional execution steps:** To increase workflow stability and reproducibility, we implemented several data-dependent conditional execution steps, as Snakemake checkpoints. These dynamic steps address the limitation of the workflow components, which are not necessarily robust to variation in the input and/or are not always necessary. By integrating actionable information from intermediate results, the execution graph is dynamically recomputed, filtering out unnecessary steps and thus ensuring a more time-effective and robust execution. The first checkpoint defined allows MosaiCatcher v2 to automatically filter out libraries flagged as low quality using both: ashleys-qc predictions and quality control measures based on reads mapping quality and coverage biases across chromosomes. The second checkpoint defined computes the average chromosomal ploidy status at the sample level (using all libraries processed) to perform haplotype phasing of heterozygous single-nucleotide polymorphisms (hetSNPs) exclusively on chromosomes that present at least a diploid status. By defining these checkpoints, we limit the chances of execution errors and ensure a smoother, more adaptive processing of inputs with a heterogeneous quality or composition.

**Read count multi-step normalization**: Strand-seq libraries, similarly to other short-read-based sequencing techniques, can be affected by various types of read coverage biases, including GC and mean–variance bias. Using a multiple step approach, we process the read data with a sequence of statistical methods: library size normalization, GC correction, and variance stabilizing transformation (VST) (detailed in the Supplementary Material). This results in a more even and unbiased read count coverage, enabling the user to have access to considerably more readable Strand-seq karyotype plots with improved characterization of SVs and copy number calling even in atypical or noisy libraries.

**Standardization, portability, and reproducibility**: To promote standardization, we implemented continuous integration/continuous development (CI/CD) control tests based on GitHub actions (Supplementary Table S1). These CI/CD tests check the different functionalities implemented into newer versions of MosaiCatcher as well as code formatting and linting. Additionally, to improve standardization, we adhered to the snakemake-workflow-catalog guidelines to comply to the “standardized usage” category of the catalog. Finally, we harmonized pipeline execution with *ad-hoc* conda environments required by the different components of the pipeline, packed into a Docker container, versioned at each new pipeline release. Taken together, MosaiCatcher v2 developed into a mature, portable, and reproducible workflow.

**SV genotyping with ArbiGent**: ArbiGent leverages strand-seq advantage in haplotype phasing for SV validation and genotyping, as well as for surveying large populations and measuring variant frequencies. This new release of Mosaicatcher features full integration of ArbiGent (Porubsky *et al.* 2022) to enable genotyping of inversions as well as all other types of SVs containing at least 500 bp of uniquely mappable sequence. Among its key functionalities, ArbiGent supports (i) fine-scaled read depth normalization to improve accuracy in complex genomic regions, (ii) methods for phase harmonization between different datasets, and (iii) a number of population-based QC metrics that can help identify spurious SV calls.

**SV functional analysis with scNOVA**: A unique feature of Strand-seq data is that they can additionally be interpreted as an epigenetic readout, a feature that is leveraged by scNOVA (Jeong *et al.* 2023), to move from the discovery of SVs in single cells to characterization of their functional consequences in the same cell. In this updated version of the MosaiCatcher pipeline, scNOVA was seamlessly integrated and can be launched using a single unified command.

**Interactive reports and downstream analysis**: To improve reproducibility and transparency, we designed HTML static web reports that can be shared easily and joined to publications, by taking advantage of Snakemake functionalities. MosaiCatcher v2 interactive reports contain all plots and information (Supplementary Table S2) required to analyze a Strand-seq experiment, from the primary analysis and quality control (FASTQC, Strand-seq QC count plots, GC analysis, cells excluded from the experiment) to the downstream analysis (single-cell SV calls plots based on different stringency criteria). Additionally, to the interactive web reports, a UCSC-genome browser custom file, as well as a IGV XML session file are automatically produced at the end of the workflow, which includes for each individual cell level Watson and Crick read counts, as well as SV calls. As presented in Supplementary Fig. S5, this allows the user a more fine-grain analysis, by investigating interactively breakpoints, orientation, or potentially associated genes and regulatory elements around the identified SVs, using the different UCSC or IGV resources.

## 3 Application

To showcase new functionalities of MosaiCatcher v2, we reprocessed—in a unique run—four cell line samples [RPE1-WT, RPE-BM510, RPE-C7, and NA20509 Lymphoblastoid Cell Line (LCL)] previously used in the original MosaiCatcher publication (Sanders *et al.* 2020) (Supplementary Table S3). Based on the full set of raw FASTQ libraries, we performed mapping against T2T-CHM13 reference assembly and automatic selection through ashleys-qc. Complete HTML report including quality control, multi-step normalization-related visualizations, and downstream analysis outputs is available as a Supplementary Data and provides an example of this more user-friendly and easy to share report that allows SV characterization at the single-cell level.

In a second run, using the same samples listed above, we demonstrate SV genotyping of 10 copy-number imbalanced SV, previously validated through WGS and emphasizing the utility of Strand-seq single-cell genotyping beyond inversions (see the Supplementary Material and Supplementary Table S4). All the events were accurately detected and no false positives nor false negatives were detected.

Finally, we constructed a “virtual mixture” of RPE cells consisting of 26 single cells from RPE-BM510 and 70 cells from RPE1-WT to demonstrate subclonal detection and functional SV impact related to subclones (Supplementary Figs S6 and S7). Following the execution of SV calling via MosaiCatcher, we established subclone groups and employed the scNOVA downstream module to analyze differences in nucleosome occupancy among the specified subclones.

## 4 Conclusion

In conclusion, MosaiCatcher v2 represents a stable, standardized, and highly user-friendly computational framework. With its key functionalities, the framework offers an end-to-end solution for Strand-seq data processing, leveraging Snakemake features.

This framework update stands to be the reference Strand-seq workflow, promoting interoperability and reusability of data within the Strand-seq scientific community. Moreover, by allowing the selection between conda and container execution, MosaiCatcher v2 fosters interoperability and reusability. Additionally, improvements in stability greatly increase efficiency, making this pipeline well suited for high-scale processing, matching the increasing amount of Strand-seq data produced in human genetics and cancer genomics, as well as SV characterization in population studies.

## Supplementary Material

btad633_Supplementary_Data

## Data Availability

The data underlying this article are available in Zenodo at the folowing links: Strand-Seq input example data part 1: https://zenodo.org/record/7696695 Strand-Seq input example data part 2: https://zenodo.org/record/7697329 MosaiCatcher v2 publication data interactive report: https://zenodo.org/record/8005968
