# Supplementary figures and images for: MosaiCatcher v2: a single-cell structural variations detection and analysis reference framework based on Strand-seq
Source: Bioinformatics. 2023 Oct 18;39(11):btad633. doi: 10.1093/bioinformatics/btad633 (PMC10628386; doi:10.1093/bioinformatics/btad633)

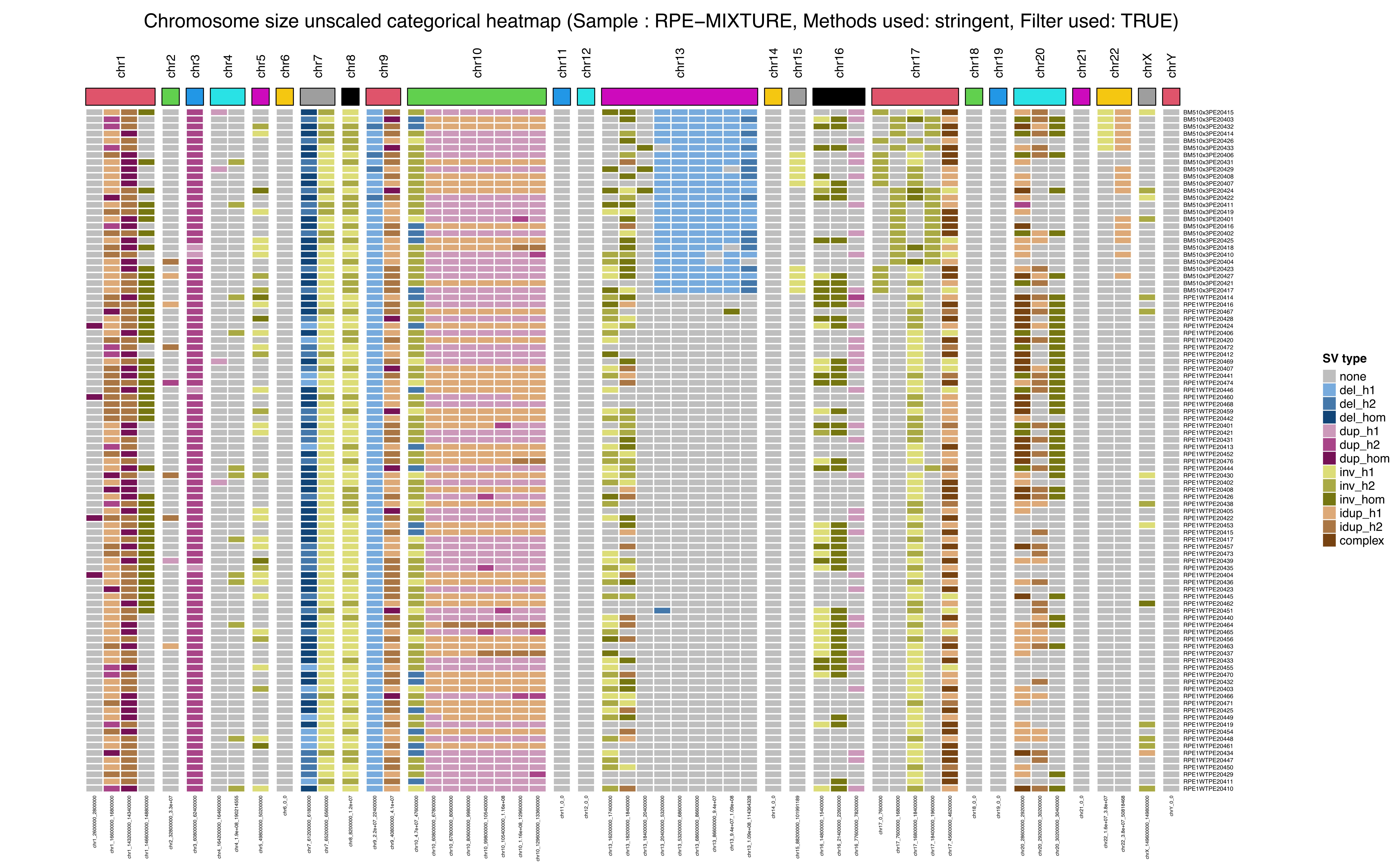

Supplement: btad633_Supplementary_Data [file btad633_supplementary_data.zip › FigS6.jpg]
